# *Brugia malayi* Glycoproteins Detected by the Filariasis Test Strip Antibody AD12.1

**DOI:** 10.3389/fitd.2021.729294

**Published:** 2021-08-29

**Authors:** Marla I. Hertz, Irene Hamlin, Amy Rush, Philip J. Budge

**Affiliations:** Division of Infectious Diseases, Department of Medicine, Washington University School of Medicine, St. Louis, MO, United States

**Keywords:** lymphatic filariasis, glycoprotein, loiasis, rapid diagnostic assays, *Brugia malayi*

## Abstract

**Background:**

Rapid and accurate prevalence mapping of lymphatic filariasis (LF) is necessary to eliminate this disfiguring and disabling neglected tropical disease. Unfortunately, rapid tests such as the filariasis test strip (FTS) for *Wuchereria bancrofti*, the causative agent of LF in Africa, can cross-react with antigens circulating in some persons infected by the African eye worm, *Loa loa*, rendering the test unreliable in eleven co-endemic nations. The intended target of the FTS is a heavily glycosylated *W. bancrofti* circulating filarial antigen (Wb-CFA). Previously, we determined that the FTS monoclonal antibody, AD12.1, which detects a carbohydrate epitope on Wb-CFA, also detects multiple *L. loa* proteins in cross-reactive sera from persons with loiasis. Since the carbohydrate epitope recognized by AD12.1 is present on glycoproteins of other parasitic nematodes, including *Brugia* species, it is unclear why reactive glycoproteins are not detected in infections with other filarial parasites.

**Methods:**

To gain a better understanding of the proteins recognized by the FTS diagnostic antibody, we used proteomics and lectin array technology to characterize filarial glycoproteins that are bound by the AD12.1 antibody using *Brugia malayi* as a model.

**Results:**

Distinct but overlapping sets of AD12 glycoproteins were identified from somatic and excretory/secretory worm products. One of the identified proteins, Bm18019 was confirmed as a secreted AD12-reactive glycoprotein by in-gel proteomics and immunoassays. Based on lectin binding patterns, *Brugia* AD12-reactive glycoproteins express glycans including core fucose, galactose, N-acetylglucosamine and galactose (β1–3)N-acetylgalactosamine in addition to the epitope recognized by AD12.1. None of the lectins that bound *B. malayi* AD12 glycoproteins had affinity for the Wb-CFA, highlighting a key difference between it and other AD12 glycoproteins.

**Conclusions:**

*B. malayi* somatic and excretory/secretory proteins are similar to *L. loa* antigens found in FTS-positive human sera, bolstering the hypothesis that circulating *L. loa* AD12 antigens result from worm tissue damage or death. The difference in glycan and protein composition between the Wb-CFA and other AD12 glycoproteins can be used to differentiate LF from cross-reactive loiasis.

## INTRODUCTION

Filarial parasites are insect-borne, tissue-dwelling, parasitic nematodes that cause debilitating diseases such as lymphatic filariasis (LF, caused by *Wuchereria bancrofti, Brugia malayi* and *Brugia timori*), onchocerciasis (by *Onchocerca volvulus*), and loiasis (by *Loa loa*). *L. loa* infection complicates disease elimination programs for LF by rendering false positive diagnostic tests, resulting in misdiagnoses (1–4), and by causing severe adverse reactions to treatments for onchocerciasis and LF (5).

Rapid diagnostic tests for Bancroftian filariasis (LF-RDT) recognize a carbohydrate epitope expressed on a high molecular weight circulating filarial antigen from *W. bancrofti* (Wb-CFA). We refer to this carbohydrate as the “AD12 epitope” (AD12e) based on its recognition by two monoclonal antibodies AD12.1 and DH6.5 (6). Based on AD12.1 binding to a glycan array, the AD12e likely contains a terminal β-d-glucuronic acid in a 1–3 linkage to other hexoses (7). AD12e is found on glycoproteins from other nematode species. However, in most other human filarial infections AD12e-containing proteins are not detected in circulation at appreciable levels. *In vitro*, AD12e glycoproteins can be detected in somatic and secreted preparations from different stages and sexes of B*. malayi, L. loa* and *Onchocerca ochengi* (6, 8, 9). Serum from baboons and immunocompromised mice experimentally infected with *L. loa* test positive by LF-RDT indicating the presence of circulating antigens (Loa CFAs) (8, 10). Lastly, LF-RDT cross-reactivity occurs naturally in a subset of loiasis infections and is associated with high microfilaria loads (1–3).

We have previously shown that cross-reactivity is due to the presence of multiple Loa CFAs (9). Understanding the nature of AD12e glycoproteins in non-Bancroftian filarial infections might prove key to developing diagnostic tests to remedy the specificity drawbacks of the current LF-RDT. We therefore catalogued AD12 glycoproteins from the model filarial organism *B. malayi* (Bm) and compared the surface glycans between brugian AD12 glycoproteins and Wb-CFA in order to identify differences between the Wb-CFA and other AD12 glycoproteins. The focus on *B. malayi* in this study stemmed from a lack of access to *L. loa* biological material. However, *B. malayi* has proven to be an apt model for *L. loa* in this case, as the AD12 glycoproteins found in *B. malayi* and *L. loa* soluble antigens share biochemical features that differ from the Wb-CFA. For example, they may contain additional N-glycans and are more susceptible to proteolytic cleavage than the Wb-CFA (7).

## MATERIALS AND METHODS

### Parasite *In Vitro* Culturing and Protein Preparation

*B. malayi* worms were obtained from the Filariasis Research Reagent Resource Center (FR3, filariasiscenter.org). To prepare whole worm lysate (*B. malayi* somatic antigens, BmSOM), 30 adult male or female worms or 10^6^ Microfilaria (Mf) were subjected to one freeze-thaw cycle, then incubated at 4°C overnight in RIPA lysis and extraction buffer (ThermoFisher) with ProteaseArrest protease inhibitors (G-biosciences). Lysates underwent mechanical disruption by pestle and then by shearing with a 25G needle with subsequent centrifugation (13500 g for 30 min). To collect excretory/secretory products (BmES), 60 adult male or female worms or 3 × 10^6^ Mf were cultured for two weeks at 37°C/5% CO_2_ in RPMI 1640 media supplemented with L-glutamine, glucose, 100 U/mL penicillin, 0.1 mg/mL streptomycin, and 0.25 μg/mL amphotericin B. The spent media was collected on alternating days by passage through a 0.4 μm cell strainer to block collection of larvae and stored at −20°C. BmES products were concentrated to 1–1.5 mL by centrifugal filtration using an Amicon Ultra 3000 MWCO filter (Millipore). In this report, BmES is derived from adult female worms unless otherwise specified. *L. loa* adult worms were a gift from Dr. Vida Dennis, Tulane University. *L. loa* soluble antigen was prepared by grinding adult worms in extraction buffer (10 mM Tris pH 8.3, 2% sodium deoxycholate, 1 mM PMSF, 1 mM EDTA, 1 mM EGTA, 25 μg/mL TLCK protease inhibitor, 15 ug/mL TPCK protease inhibitor) and collecting the soluble fraction. *Onchocerca volvulus*, *Acanthocheilonema viteae*, *Ascaris suum*, and *Paragonimus westernmani* lysates were a gift from P. Fischer and G. Weil at Washington University in St. Louis. *Caenorhabditis elegans* N2 worm lysates were a kind gift from Z. Pincus at Washington University in Saint Louis. Human sera from persons with Bancroftian LF was sourced from banked, de-identified human samples from prior studies (11, 12).

### Antibodies

DH6.5 and AD12.1 are monoclonal mouse IgM antibodies, initially generated against *Dirofilaria immitis* antigens, which both recognize the same carbohydrate epitope (6). A mouse IgM specific to lipopolysaccharide (clone 11E10, Southern Biotech) served as an isotype control. To generate antibodies against the *B. malayi* protein Bm18019, we immunized rabbits with the synthetic peptide c-DFSTNYDIRTESEWNSYAK (LifeTein) per a standard 70-day immunization protocol, and serum was affinity-purified to produce polyclonal immunoglobulin (Ig) concentrates (LifeTein). Horseradish peroxidase was conjugated to the AD12.1 antibody (AD12-HRP) and used at a concentration of 0.3 μg/mL.

### Immunoprecipitation

Monoclonal antibody DH6.5, mouse IgM isotype control, anti-Bm18019 polyclonal Ig or normal rabbit Ig (RbN) control antibodies were conjugated to Affi-gel 10 beads (Bio-Rad) according to manufacturer’s protocol. Conjugated beads were stored as a 50% slurry in PBS. Conjugated beads (100 μL) were mixed with either (i) human sera (as a source of Wb-CFA), (ii) 80 μg of BmSOM, or (iii) 80 μg of BmES in 1 ml total volume then rocked overnight at 4°C. For human sera and lysate, beads were washed four times with cold PBS. For BmES, more stringent wash conditions were required due to the lack of detergent during BmES preparation. BmES product was washed four times with cold PBS, four times with high salt detergent buffer, MIB-T [5 mM HEPES, 1 M NaCl, 1 mM EDTA, 1 mM EGTA, 10 mM NaF, 2.4 mM NaVO_4_, 0.5% Triton-X-100 and Pierce protease inhibitor cocktail mini tablet (ThermoFisher)], followed by two washes in cold PBS to remove the detergent. After washing, antigen-coated beads were suspended in 1X NuPAGE LDS sample buffer (Invitrogen) and incubated at 95°C for five minutes to release bound antigens.

For Bm18019 experiments, immunoprecipitation of five μg BmES with 50 μL anti-Bm18019-bead conjugate were incubated overnight at 4°C followed by washing in MIB-T buffer and elution in 1X LDS sample buffer as written above. Immunoprecipitates were assayed by western blot and/or submitted for proteomics analysis as described below. Reagents for immunoprecipitation, immunoblot and ELISA experiments were purchased from Sigma-Aldrich, unless otherwise noted.

### Polyacrylamide Gel Electrophoresis, Silver Stain, and Western Blot Analysis

Proteins were boiled in 1X NuPAGE LDS reducing sample buffer then resolved by SDS-PAGE using a 4–12% bis-tris NuPAGE gel (Invitrogen). Silver staining of gels was performed with the FOCUS FAST silver staining kit (G-Biosciences) according to the manufacturer’s instructions. For western blots, proteins were transferred to nitrocellulose membranes (Amersham), incubated with blocking buffer [5% non-fat dry milk (Nestle Carnation) in phosphate buffered saline with 0.05% Tween-20 (PBS-T)] for one hour, then incubated with AD12-HRP (1:3000 dilution in blocking buffer) for one hour at room temperature. Membranes were washed three times in PBS-T and incubated with Clarity Western ECL substrate (Bio-Rad) for chemiluminescence detection using a ChemiDoc imager and Image Lab 5.2.1 software (Bio-Rad) or in some cases as specified, developed with the colorimetric substrate CN/DAB (3,3’-Diaminobenzidine, Pierce).

### Liquid Chromatography-Electrospray Ionization- Tandem Mass Spectrometry

Immunoprecipitation samples were prepared for LC-ESI-MS/MS using a Q-Exactive Plus Hybrid Quadrupole-Orbitrap Plus mass spectrometer (ThermoFisher Scientific) coupled to an EASY-nanoLC 1000 system (ThermoFisher Scientific) as described previously (9). The resulting MS spectra were converted to Mascot generic format (MGF) files using Proteome Discoverer v2.1.0.81. MGF files were submitted for peptide identification based on the *B. malayi* genome, Bmal 4.0 (13) [downloaded from WormBase ParaSite (parasite.wormbase.org)]. Peptide hits were cross-referenced against contaminant databases: ENSEMBL for Human contaminants (Homo_sapiens.GRCh37.72 ENHU, version June 2016) and the cRAP database version (2012.01.01) for common contaminating peptides (http://www.thegpm.org/crap/). Qualifying peptides had a less than 1% false discovery rate and were absent from the combined human and cRAP databases. Proteins were considered true hits if more than one unique peptide was detected by mass spectrometry.

For in-gel proteomics, gel bands were cut into four 1 × 1 mm plugs and transferred to a 96 well plate. Gel plugs were dehydrated by the addition of acetonitrile (100 μL) to each well and incubated at room temperature for 10 min on a nutator. The supernatants were removed, the dehydration step was repeated, and the gel plugs were air dried for 30 min. Disulfide bonds were reduced with 50 μL of 10 mM DTT in 100 mM Seppro ammonium bicarbonate buffer (ABC buffer, Millipore), pH 8.0 for 30 min at 56°C, followed by alkylation with 50 μL of 55 mM IAM in 100 mM ABC buffer for 20 min at room temperature in the dark. Plugs were dehydrated twice with acetonitrile as before. Gel plugs were rehydrated in digestion buffer containing either chymotrypsin (0.025 μg/μL in LC-MS-grade water) or trypsin (0.025 μg/μL in 100 mM ABC buffer, pH 8.0) and incubated for 1 hour at room temperature on the nutator. An additional 0.25 μg of digest enzyme was added and the sample was incubated overnight. Peptides were extracted twice from the plugs by incubating with 20 μL of 50% MeCN with 5% (vol/vol) formic acid in water at 37°C for 30 min. Peptides were analyzed according to the solution-based proteomics described above.

### Bioinformatics Analysis

Blast2Go software was used to assign Gene Ontology (GO) terms to the *B. malayi* immunoprecipitation (IP) combined dataset and to the *B. malayi* proteome to build a reference database (14). NetOGlyc 4.0 was used to predict O-glycosylation sites in the hits from the mass spec analysis (15). These results were compared with the O-glycosylation prediction of 500 randomly selected proteins from the *B. malayi* genome to estimate enrichment of O-glycoproteins in the IP and p-values were calculated by binomial distribution. The same parameters were used to predict N-glycosylation sites with NetNGlyc 1.0 (16).

### Lectin Array

A 40-Lectin array (GA-Lectin-40, RayBiotech) was used according to the manufacturer’s standard protocol. The array was probed with BmSOM 2 μg/μl, BmES 117 ng/μl, or DH6.5 immunoaffinity purified Wb-CFA from 2 mL pooled serum eluted from beads by heating at 100°C in PBS for 5 minutes (11). Cy-3 was directly conjugated to the AD12.1 antibody using the Cy3^®^ Conjugation Kit (Fast) - Lightning-Link^®^ (Abcam) according to the manufacturer’s protocol. Cy3 conjugated antibody was used at a 2 μg/mL dilution as a detection reagent. The array slide was shipped to RayBiotech and scanned using the GenePix optical scanner.

### ELISAs

#### Filarial Antigen and Lectin Capture ELISAs

Detection of Wb-CFA by sandwich ELISA was performed as previously described (6, 9). For antigen capture using lectins, wells were similarly coated overnight at 37°C with 20 μg/mL of one of the following lectins in 0.6 M carbonate buffer, pH 9.6: Jacalin, wheat germ agglutinin (WGA), peanut agglutinin (PNA), *Aleuria aurantia* lectin (AAL, Vector Laboratories), *Dolichos biflorus* agglutinin (DBA, GlycoMatrix), *Artocarpus integrifolia* lectin (AIA, GlycoMatrix), and *Psophocarpus tetragonolobus* lectin II (PTL, GlycoMatrix). After coating, wells were washed with phosphate buffered saline (PBS) containing 0.05% Tween-20 (PBS-T) then blocked with 200 μL 5% fetal calf serum in PBS-T for one hour at 37°C. BmSOM or human sera [heated at 56°C for 30 min and EDTA treated to dissociate immune complexes as previously described (17)] was then added and incubated for two hours at 37°C. Wells were washed three times with PBS-T and incubated with AD12-HRP for one hour at 37°C. After a final round of PBS-T washes, 100 μL O-phenylenediamine dihydrochloride (OPD, ThermoScientifc) substrate was added and allowed to develop for ten minutes. The reaction was stopped by adding 30 μL of 4 M sulfuric acid, and absorbance was read at 492 nm. For sequential depletion of antigens, this standard format was modified by adding 0.5 μg/mL BmSOM to lectin-coated wells, incubating overnight at 37°C, then transferring the supernatant from each well to a another lectin-coated capture well for a second overnight incubation at 37°C. Finally, the supernatant from the second incubation was added to a DH6.5-coated well and incubated for one hour at 37° C to capture any remaining antigen. Captured antigen in all wells was detected using AD12-HRP as described above.

#### Anti-Bm18019 ELISA

To detect Bm18019 in BmES, wells were coated overnight at 37°C with 20 μg/mL of anti-Bm18019 polyclonal Ig in 0.6 M carbonate buffer, pH 9.6. Wells were washed and blocked then incubated for one hour at 37°C with 0–2 μg of BmES. Captured antigen was detected using AD12-HRP as described above.

### Enzyme Treatment

*PNGase F*. Four micrograms of BmES were digested with PNGase F (New England Biolabs) under denaturing conditions for one hour at 37°C following the manufacturer’s protocol. Two micrograms of RNase B were used in a positive control assay to confirm enzyme activity (New England Biolabs). *OSGE*. Lyophilized O-sialoglycoprotein endopeptidase purified from *Mannheimia haemolytica* (OSGE, CedarLane Labs) was reconstituted to 2.4 mg/mL in deionized water. Two micrograms of BmES were cleaved with 3 μl of OSGE at 37°C for 0–4 hours. The reaction was stopped by boiling at 100°C in 1X NuPAGE LDS sample buffer (Invitrogen). Digests were analyzed by immunoblot or silver stain.

## RESULTS

### AD12e Decorates Many Nematode Proteins

**FIGURE 1 |:**
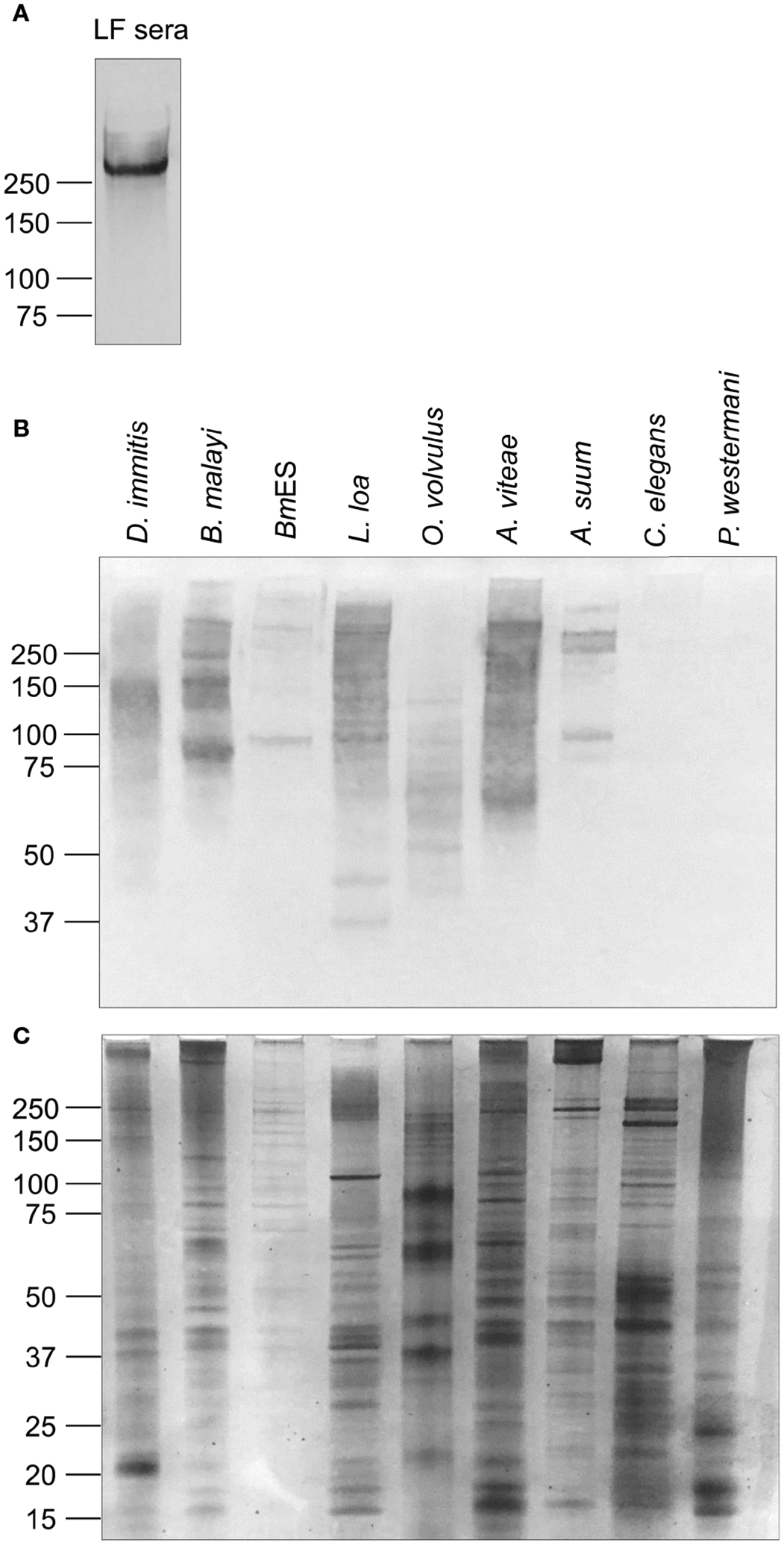
The glycan epitope detected by monoclonal antibody AD12.1 is present on multiple nematode proteins. **(A)** Pooled sera from *W. bancrofti*-infected patients in Sri Lanka were incubated overnight with Affi-Gel 10 beads conjugated to monoclonal antibody DH6.5. Bound Wb-CFA was eluted and detected by AD12-HRP by western blot. One microgram of each of the following antigen preparations: *Dirofilaria immitis*, *Brugia malayi* total lysate*, Brugia malayi* secreted antigens (BmES), soluble antigens from *Loa Onchocerca volvulus*, *Acanthocheilonema viteae, Ascaris suum*, *Caenorhabditis elegans* and *Paragonimus westermani* were analyzed by **(B)** CN/DAB colorimetric western blot using AD12.1 antibody or **(C)** silver stain.

It is well established that sera from persons with Bancroftian filariasis have a single circulating filarial antigen, the Wb-CFA, which is ~250 kDa in size and is abundantly decorated with the AD12e glycan epitope (Figure 1A). By western blot, AD12e was detected on multiple antigens of varying molecular weights in extracts from several filarial and non-filarial nematodes, but not in the trematode *Paragonimus westermani* (Figure 1B). A faint AD12e signal was observed with extract from the free-living nematode, *Caenorhabditis elegans*; when a higher concentration of *C. elegans* lysate was analyzed, three distinct bands at 50 kDa, 100 kDa and greater than 250kDa were detected (Figure S1). A silver stain of the protein extracts served as a loading control (Figure 1C).

### AD12e Glycoproteins From BmSOM and BmES Are Similar to Cross-Reactive Antigens From Loiasis Sera

**FIGURE 2 |:**
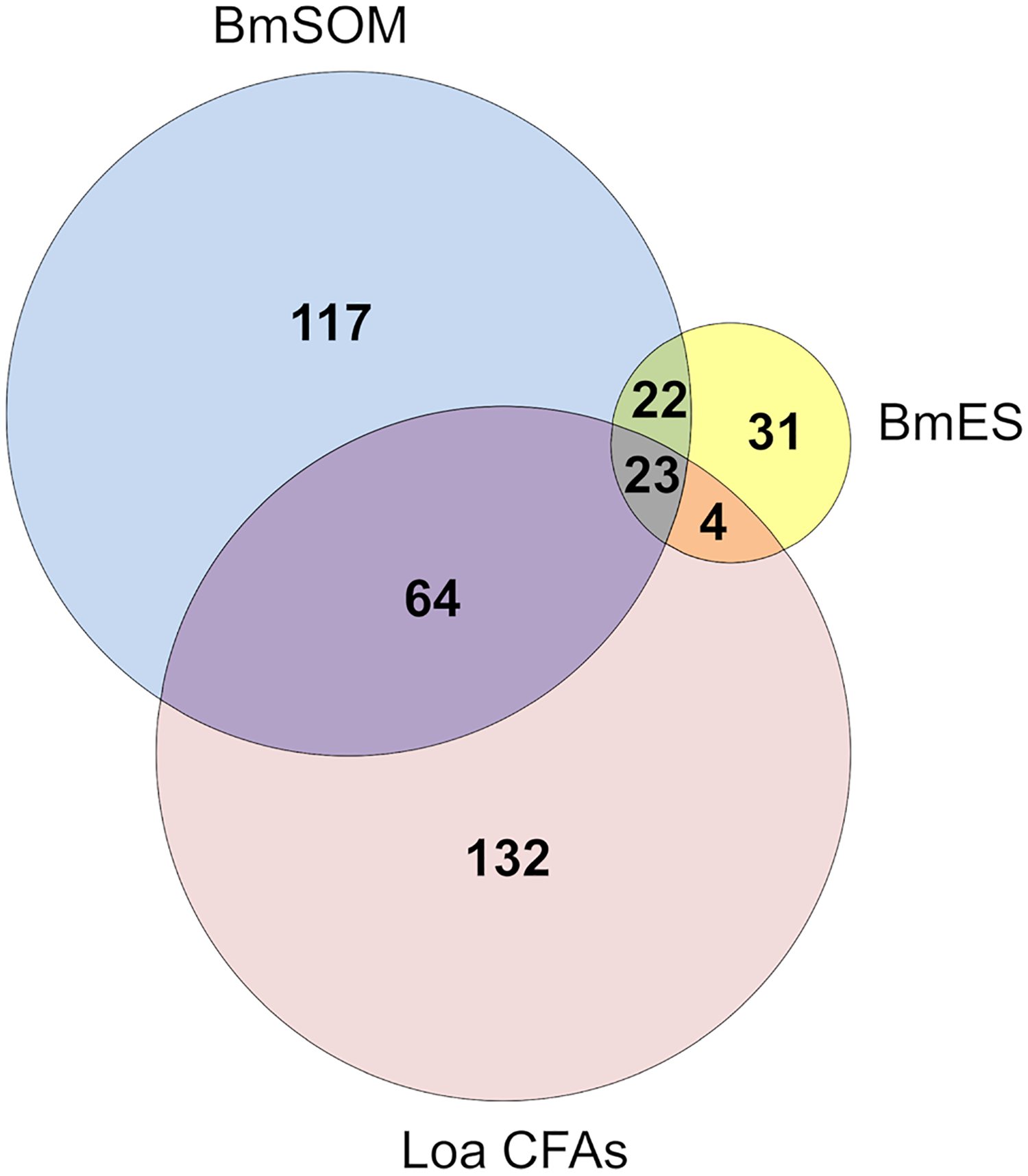
Mass spectrometry results. Two hundred and fifty four proteins were identified from DH6.5 immunoaffinity purifications of Brugian protein preparations (222 from BmSOM and 77 from BmES). The most abundant hits from each category are detailed in Table 1 with a complete list found in Table S1. The 223 *L. loa* proteins from cross-reactive loiasis sera (Loa CFAs) were previously published (9).

The function of the AD12e glycan and glycoproteins that contain it are unknown. To gain insight into the types of proteins containing AD12e, we cataloged the AD12e-containing glycoproteins in adult female somatic (BmSOM) or excretory/secretory (BmES) product from *B. malayi*. Proteins were immunoaffinity purified with DH6.5 or a mouse IgM isotype control and analyzed by liquid chromatograph-tandem mass spectrometry. Spectra from BmSOM matched to 1784 peptides in 226 *B. malayi* proteins, after excluding proteins also detected in the isotype control. A similar analysis of BmES products yielded 560 unique peptides assigned to 80 proteins, 31 of which were not detected in BmSOM. Table 1 lists the most abundant proteins from each sample; a complete list of proteins detected is available in Table S1. Two hundred and seven (79.3%) of the 261 combined BmSOM and BmES protein hits were predicted by NetOGlyc 4.0 (18) to have O-linked glycosylation sites (O-glycosite, Table 1), compared to 73.9% (95% CI: 70% – 78%) of a random set of 500 *B. malayi* proteins. NetNGlyc 1.0 software (19) predicted that 138 (52.9%) of the proteins we identified contained N-linked glycosylation sites (N-glycosites) compared to 23.6% (95% CI: 20% - 28%) of the 500 randomly selected *B. malayi* proteins. Thus, the immunoprecipitation enriched for both O- and N- linked glycoproteins (respective p-value based on binomial distribution are 0.008 and 1.4 × 10^−24^). The list of brugian AD12 glycoproteins was compared to *L. loa* circulating antigens (Loa CFAs) to attempt to define the origin of RDT positive antigens in loiasis. When the Brugian proteins were compared to Loa CFAs, 88 direct homologs of the 233 Loa CFAs (38%) were detected in *B. malayi* (Figure 2). Thus, the filarial parasites have overlapping repertoires of AD12 glycoproteins and Loa CFAs include both somatic and secreted antigens.

Gene ontology (GO) enrichment analysis of the combined BmSOM and BmES hits, compared to the *B. malayi* genome (13) indicates that Brugian AD12 glycoproteins are enriched in cytoplasmic, non-membrane bound organelle, and cytoskeletal compartments; oxidoreductase and hydrolase activity; and in reproductive and early developmental processes (Tables S1, S2). Strikingly, all but one (nucleoside-triphosphatase activity) of the GO terms enriched in *B. malayi* were also enriched in the previous analysis on Loa CFAs (9) indicating a conserved function.

### Identification of a Prototype BmES AD12 Glycoprotein

**FIGURE 3 |:**
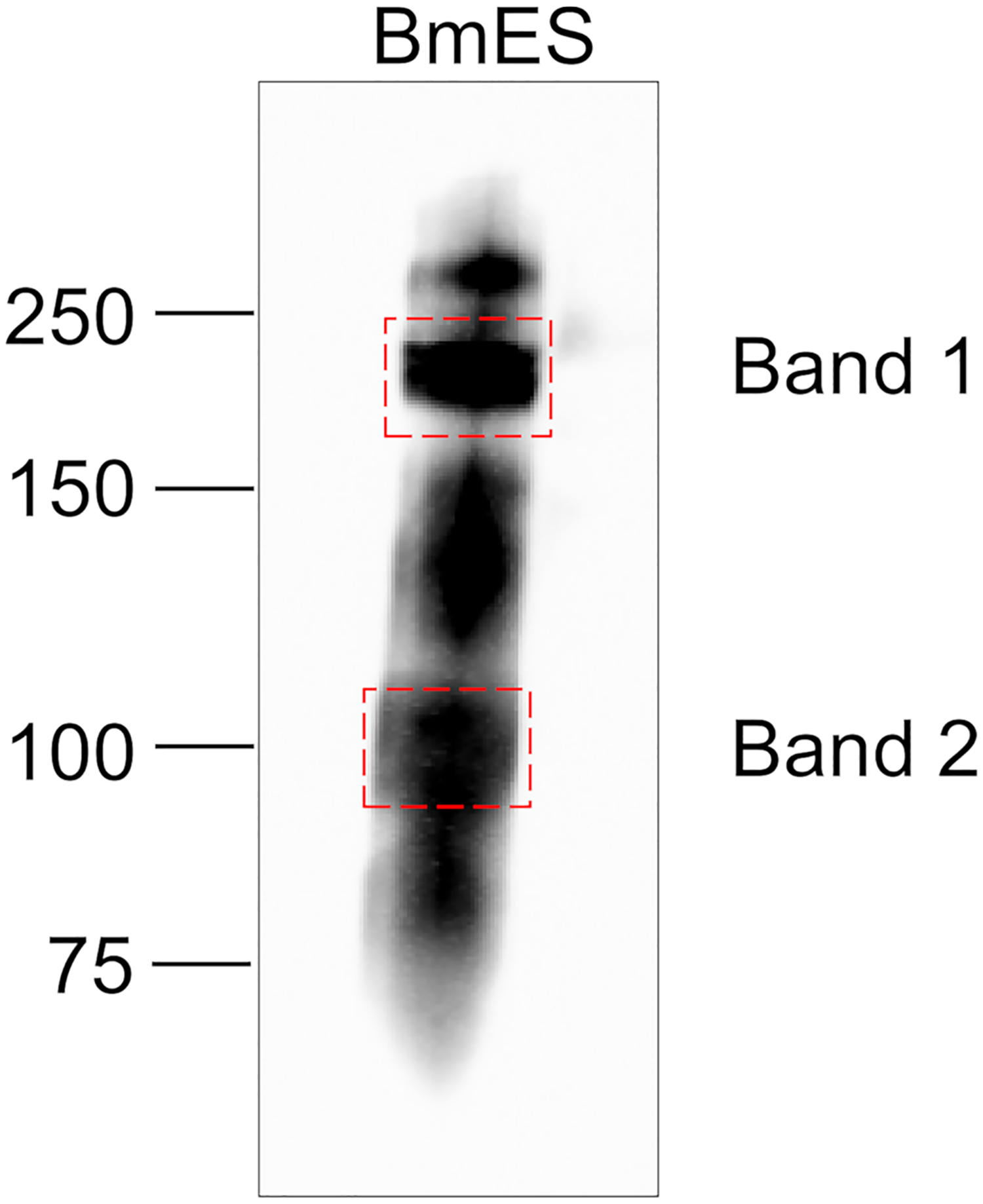
Gel slices analyzed by LC/MS/MS. 10 μg of BmES from adult female *B. malayi* were immunoprecipitated with DH6.5-conjugated Affi-gel beads. Bound proteins were eluted in SDS sample buffer, resolved using a 4–12% Bis-Tris gel and immunoblotted with AD12-HRP to identify regions of interest. Bands indicated by dashed boxes were excised for proteomic analysis.

To identify a single model glycoprotein to study AD12 glycan structure and function, we excised two predominant AD12.1-reactive bands from BmES for proteomic analysis (Figure 3). Band 1 contained thirty-eight spectra and Band 2 contained thirteen spectra that were assigned to brugian peptides. Two or more unique peptides were detected from four proteins in Band 1 and three proteins in Band 2 (Table 2). One protein from Band 2 was selected for further analysis since it was the only detected protein that migrated at a larger than predicted molecular weight, suggesting it might be heavily glycosylated.

The amino acid sequence of Bm18019 indicates that it is a mucin-like protein, and is primarily composed of eleven copies of a repeat element, rich in serine and threonine, of which 106 are in good context to serve as o-glycosylation sites (15). MS peptides mapped to the unique amino and carboxy termini (Figure S2).

**FIGURE 4 |:**
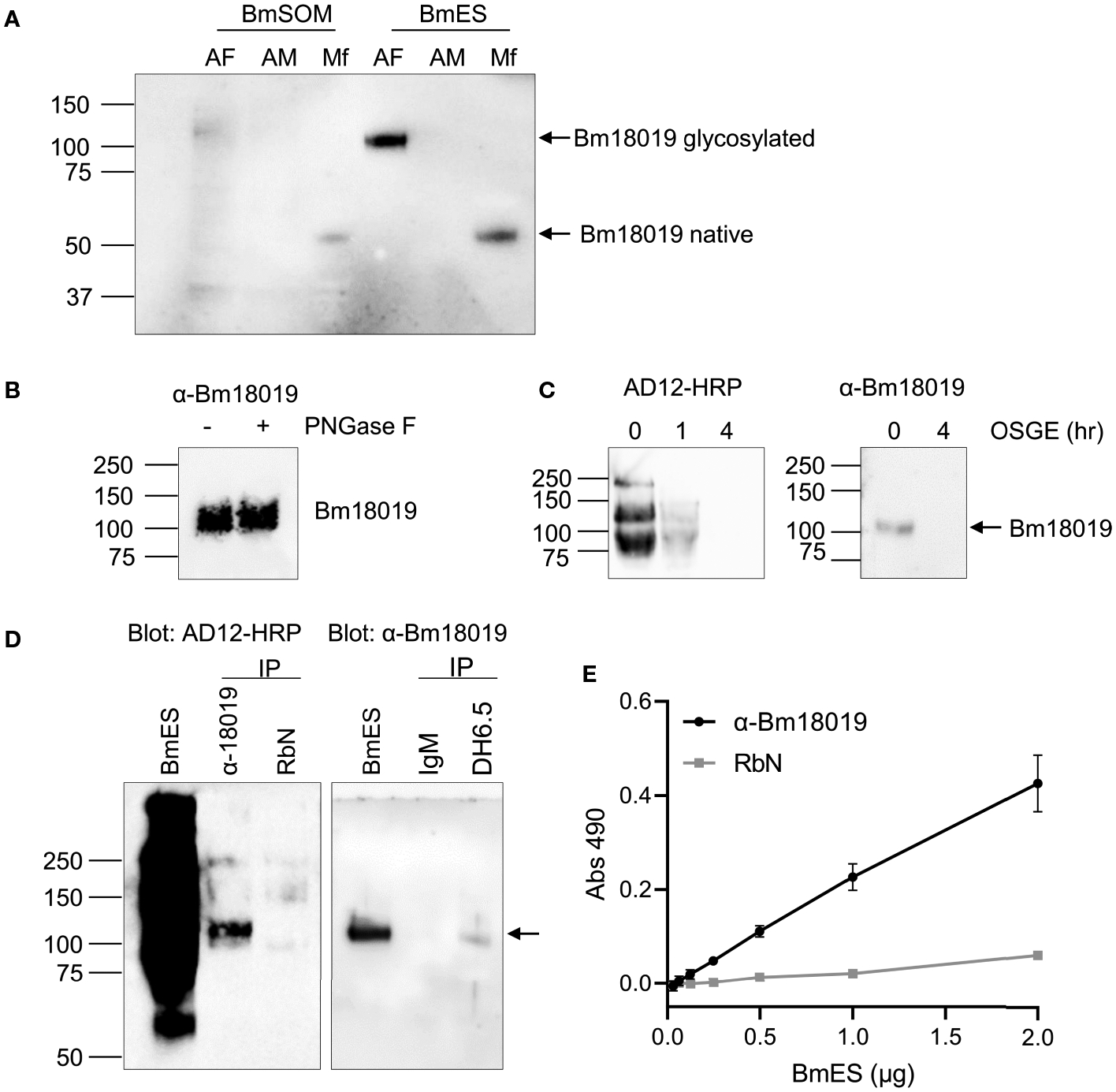
Characterization of Bm18019 expression and glycosylation. **(A)** Western blot of *B. malayi* antigen preparations probed with an anti-Bm18019 polyclonal Ig. Each lane contains 2 μg of protein purified from BmSOM or BmES from AF, adult female; AM, adult male; Mf, microfilaria. The predicted molecular weight of Bm18019 polypeptide is 53 kDa. **(B)** PNGase F digestion of 4 μg BmES were analyzed by western blot using anti-Bm18019. Activity of PNGase F was confirmed by a Coomassie stain of a control reaction using RNase B as a substrate (Figure S3). **(C)** Bm18019 is sensitive to the mucinase OSGE. 2μg of BmES was incubated with OSGE for 0–4 hours and then probed with AD12-HRP (*left*) or anti-Bm18019 (*right*). **(D)** Reciprocal immunoprecipitation confirms that Bm18019 is detectable by the AD12.1 antibody. Immunoprecipitation of 5 μg BmES with antibody conjugated Affi-gel beads were examined by western blot. An isotype matched control IP reaction was performed with normal rabbit sera (RbN) and mouse IgM-conjugated beads. **(E)** Sandwich ELISA demonstrates that native Bm18019 is concurrently bound by anti-Bm18019 and AD12.1. Plates were coated with anti-Bm1801or RbN control. A serial dilution of BmES was used as a source of antigen followed by detection with AD12-HRP.

Polyclonal immunoglobulin, anti-Bm18019, was generated against a peptide in the C-terminus and used to further characterize the protein. Bm18019 was detected as a 100 kDa band in adult female BmES with a faint signal in in BmSOM (Figure 4A). The molecular weight corroborated the proteomic results and suggested that the protein is heavily post-translationally modified. Interestingly, a band that migrates at the predicted size for the native protein (53 kDa) was strongly detected in Mf-ES and weakly detected in Mf-SOM (Figure 4A). Together, these data show that Bm18019 is a secreted protein with sex and stage specific expression.

To characterize the glycosylation properties of Bm18019, adult female BmES was treated with PNGase F, an enzyme that cleaves N-glycans, and O-sialoglycoprotein endopeptidase (OSGE), a mucinase. Bm18019 was not cleaved by PNGase F (Figure 4B). Enzymatic activity was validated by the positive control reaction with RNase B as a substrate (Figure S3). Like all AD12 glycoproteins in *B. malayi*, Bm18019 was completely degraded by OSGE after a 4-hour incubation (Figure 4C). Sensitivity to OSGE and resistance to PNGase F is characteristic of O-linked mucins.

To confirm the presence of the AD12 glycan epitope on Bm18019, reciprocal immunoprecipitation reactions were conducted (Figure 4D). Precipitation of BmES with the DH6.5 antibody contained Bm18019 and conversely, precipitation with anti-Bm18019 was positive in an AD12 immunoblot. A lack of signal when immunoprecipitation reactions were performed with isotype control antibodies validated the specificity of the assay. In an ELISA format, we were able to capture Bm18019 with the anti-peptide antibody, followed by detection with HRP-labeled AD12.1, suggesting that both AD12 and anti-Bm18019 could bind the intact native Bm18019 simultaneously (Figure 4E). Taken together these results confirm that Bm18019 expresses the AD12 reactive glycan on its surface and that at least some of the AD12.1 binding sites on Bm18019 are not masked by binding of the anti-peptide poly Ig. The OSGE sensitivity of all Brugian AD12 glycoproteins and this preliminary characterization of a specific, mucin-like AD12 glycan, supports the hypothesis that the AD12 glycan is O-linked and present on mucin-like glycoproteins.

### Lectin Binding Profile of AD12 Glycoproteins

**FIGURE 5 |:**
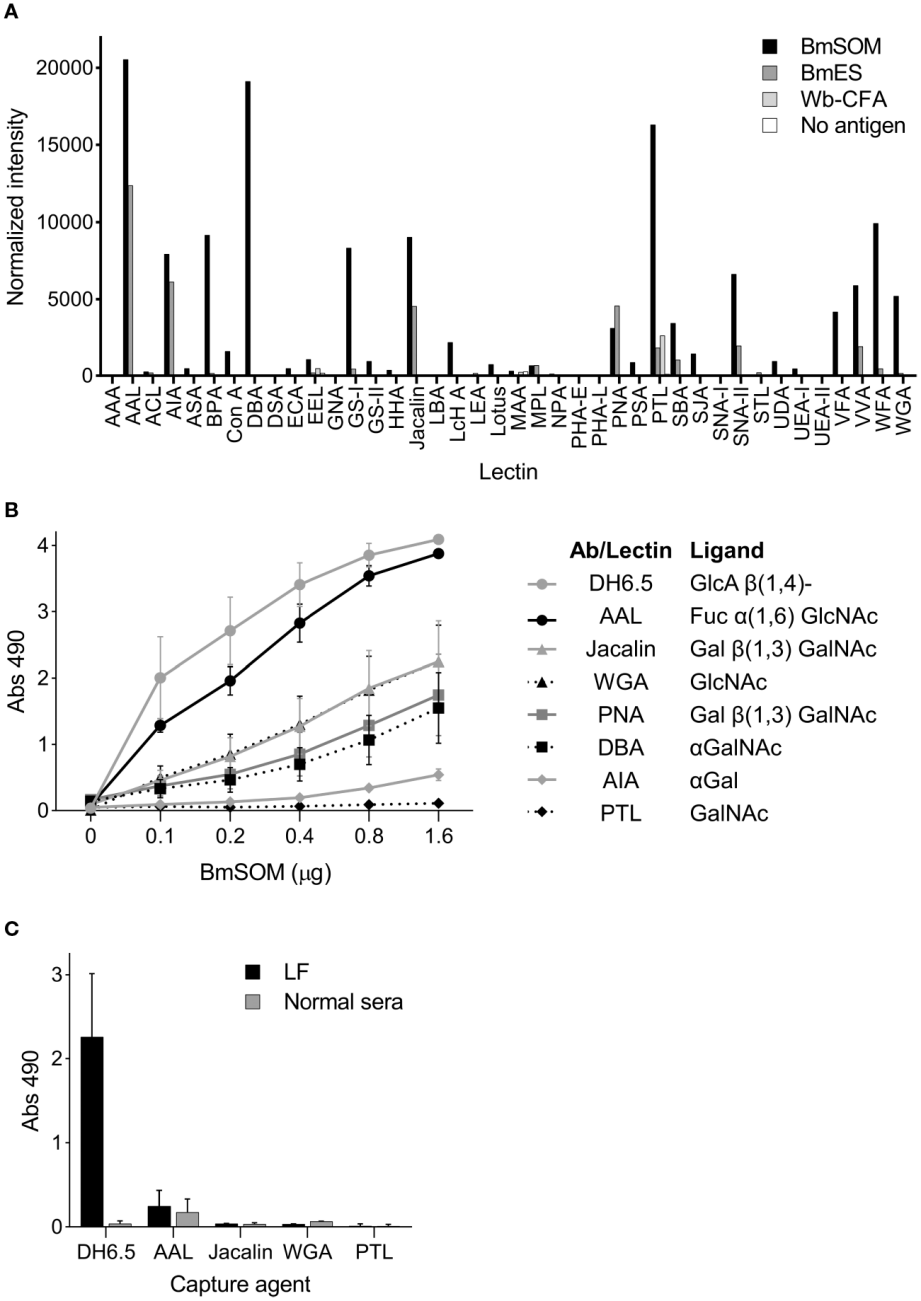
Lectins bind to AD12 glycoproteins from *B. malayi* but not to the Wb-CFA **(A)** The RayBiotech 40 lectin array was incubated with 2 μg/μl BmSOM, 117 ng/μl BmES, Wb-CFA immunoprecipitated from human sera, or an antigen free control. Captured glycoproteins were detected with a Cy-3 conjugated AD12.1 antibody. Normalized signal intensity was averaged from duplicate wells. **(B, C)** Lectin capture assay. **(B)** A two-fold serial dilution of BmSOM was incubated in wells individually coated with DH6.5 or lectins. Bound glycoproteins were detected with AD12-HRP. Error bars represent standard deviation for three independent experiments performed in triplicate. **(C)** Immobilized DH6.5 or lectins were incubated with human sera pooled from 26 LF subjects (Black bars) or 12 nonendemic subjects (gray bars). Bound antigen was detected with AD12-HRP. Error bars represent standard deviation for two independent experiments performed in triplicate. AAL, aleuria aurantia lectin; WGA, wheat germ agglutinin; PNA, peanut agglutinin; DBA, dolichos biflorus agglutinin; AIA, artocarpus integrifolia lectin; PTL, psophocarpus tetragonolobus lectin II. Fuc, fucose; GlcNAc, N-acetylglucosamine; Gal, galactose; GalNAc, N-acetylgalactosamine.

To compare the surface glycans on the Wb-CFA and *B. malayi* AD12 glycoproteins and identify lectins that uniquely bound one or the other we performed a lectin array. Multiple lectins bound to AD12 glycoproteins from BmSOM and a subset bound to BmES (Figure 5A). The strongest binders were *Aleuria aurantia* lectin (AAL, which binds fucose), *Dolichos biflorus* agglutinin (DBA, binds α-linked *N*-acetylgalactosamine, αGalNAc) and *Psophocarpus tetragonolobus* lectin I (PTL, binds *N*-acetylgalactosamine, GalNAc). The raw lectin array data are available in Table S3. The Wb-CFA did not interact strongly with any lectin on the array. The absence of lectin binding to the Wb-CFA agrees with prior work which noted a lack of binding by concavalin A, soybean lectin, wheat germ agglutinin, and Ricin II and III lectins to Wb-CFA (6). The signal intensity in the ‘no antigen’ control sample indicates that lectin binding to glycans present on the detection antibody was negligible.

Using an in-house ELISA assay we confirmed that six of the seven most active array lectins bound to AD12e-containing proteins in BmSOM and none bound to the Wb-CFA (Figures 5B, C). PTL was inactive in the in-house ELISA. Thus, based on the lectin binding profiles, AD12 glycoproteins in BmSOM may contain fucose(α1–6)GlcNAc or fucose(α1–6)LacNAc, T-antigen (Galβ1–3GalNAc), GlcNAc, and αGalNAc either on the same glycan chain as the AD12e or at an alternate glycosite. For Wb-CFA, such moieties are either lacking, or are masked.

### Evaluation of Lectins as a Sink to Deplete AD12 Glycoproteins

**FIGURE 6 |:**
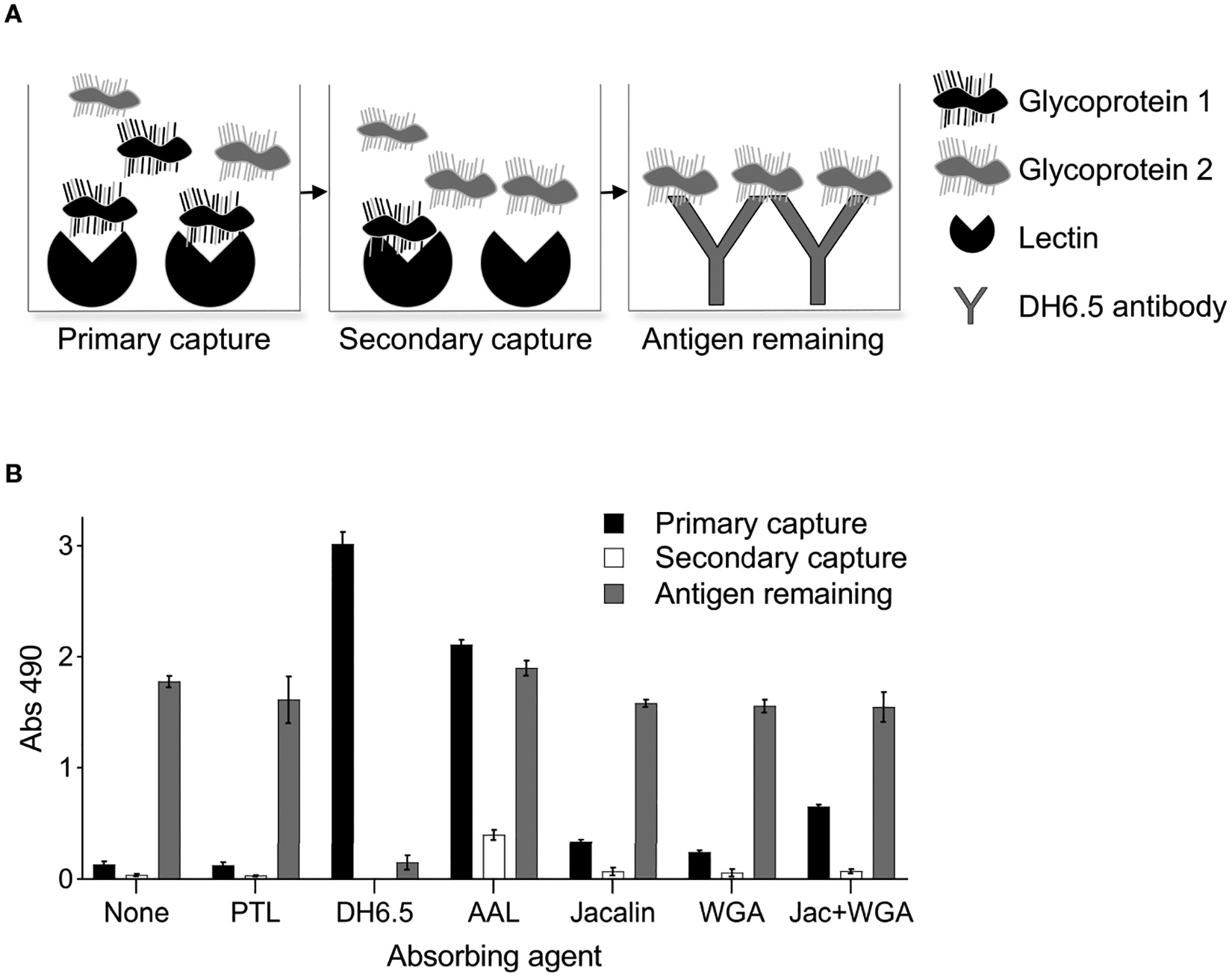
Lectin depletion of AD12 glycoproteins. **(A)** Experimental setup illustrated by a hypothetical example with two AD12 glycoproteins, one of which is depleted by the lectin (black) and the other is not (gray). 50 ng of BmSOM (at 0.5μg/mL) was incubated for 24 hours with either DH6.5 or various lectins (primary capture). The unbound fraction was incubated in a second well containing the same lectin as in the primary capture to assess depletion by the absorbing agent (secondary capture). Finally, the unbound fraction was incubated with DH6.5 to measure the remaining AD12 glycoproteins in the sample (antigen remaining). **(B)** Experimental results with BmSOM. Results displayed are an average calculated from two independent experiments performed in triplicate. Error bars depict standard deviation from the mean. PTL, *Psophocarpus tetragonolobus* lectin II; AAL, *Aleuria aurantia* lectin; WGA, wheat germ agglutinin.

Having failed to identify a lectin that could selectively capture Wb-CFA, we next assessed whether the BmSOM-reactive lectins could functionally deplete cross-reactive AD12 glycoproteins. Theoretically, lectin-depletion of cross-reactive antigens might improve the specificity of LF diagnostic tests. We therefore developed a depletion ELISA, which involved preabsorbing with a lectin and then measuring the amount of remaining glycoprotein by capture with the DH6.5 antibody. Figure 6A outlines the procedure using two hypothetical proteins, one which binds to the immobilized lectin (black) and one which does not (gray). The total amount of AD12 glycoproteins captured at each step was determined by detection with AD12-HRP. Negative controls with no absorbing agent or with PTL lectin, which in our assay did not recognize AD12e-containing proteins, illustrates the amount of antigen that remained by the end of the experiment and accounts for sample loss during transfer steps and minimal spurious binding to the plate surface. This strategy was validated by demonstrating that complete depletion was achieved in the primary capture event with DH6.5 as the absorbing agent (Figure 6B). Lectins AAL, Jacalin and WGA were able to absorb AD12e-containing proteins from BmSOM. A 24-hour incubation was sufficient to capture all available ligands per lectin as illustrated by a lack of signal in the secondary capture wells (white bars, Figure 6B). However, lectin depletion did not reduce the total signal in the final antibody capture. Combining Jacalin and WGA lectins did not increase the efficiency. Thus, preabsorption with these lectins was not robust enough to deplete the total pool of AD12 glycoproteins. Interestingly, with AAL, the total amount of AD12e bound, combined with the amount unbound, exceeded the amount of AD12e bound by the DH6.5 positive control. This likely reflects competition for AD12e binding between DH6.5 (the capture antibody) and AD12.1 (the detecting antibody), which both bind AD12e. Capture by DH6.5 will block some AD12e binding sites, whereas capture by a lectin may leave all AD12e sites unblocked and available for AD12.1 detection.

## DISCUSSION

Improvement of diagnostic tests for filarial infections is a high priority for the elimination of neglected tropical diseases. We report here some of our observations about the epitope AD12e, which is the target of current diagnostic tests for Bancroftian filariasis. Our key observations include: (1) AD12.1-reactive glycans are produced by all filarial worms tested to date, and by distantly related nematodes including *Ascaris suum* and *C. elegans*. (2) In *B. malayi*, AD12.1-reactive epitopes are on both somatic and ES glycoproteins, as has been previously shown for *L. loa* (9). (3) *B. malayi* AD12e glycoproteins, like those of *L. loa*, and unlike Wb-CFA, are susceptible to OSGE degradation. This includes a 100 kDa glycoprotein, Bm18019, with mucin-like characteristics. (4), In contrast to Wb-CFA, many *B. malayi* AD12e glycoproteins contain glycans recognized by commercially available lectins. However, no single lectin appears to capture all AD12e glycoproteins from *B. malayi*, suggesting that the lectins tested are binding to non-AD12glycans that co-decorate AD12e glycoproteins.

This report further establishes the AD12 epitope as a common glycan motif on nematode glycoproteins and the number of proteins decorated with AD12e appears to have expanded in filarial worms compared to worms with free-living stages. The role AD12e plays in filarial biology remains an open question but there is evidence that it is involved in reproduction and development. Previous studies demonstrated that adult female worms are the main producers of AD12e which is localized to the reproductive tissues (6). Our work has shown that proteins that contain AD12e are enriched in reproductive and development functional terms.

This study also characterized a specific AD12 glycoprotein, Bm18019. Bm18019 had sex and stage specific expression pattern consistent with earlier transcriptomic studies in *B. malayi*. Transcription profiling studies categorized Bm1_15985 (an earlier identification number for Bm18019), as a differentially expressed gene clustered with other genes involved in reproduction and highly expressed in adult female worms and eggs. The gene was also transcribed to a lesser extent in adult males and immature worms but not detected in the Mf to L4 stages (18, 20). Our results showing positive protein expression in Mf contradict these transcription analyses. It is possible that the gene expression was missed in the transcriptome microarrays used in the earlier studies. Alternatively, Bm18019 may be produced by the adult female worm and remain associated with the Mf. Lastly, off target detection by the novel antibody generated in this study cannot be ruled out. Proteomic evidence for Bm1_15985 is scarce; it was not detected in BmES or BmSOM (19, 21–23). However, Bm1_15985 was detected when proteomic analysis was performed on three separated body compartments (body wall, reproductive tract, and digestive tract) (24). It is possible that o-glycosylation masked peptide detection in the earlier proteomic studies of complex mixtures. Interestingly, orthologues to the Bm18019 gene have only been identified in the parasites that cause lymphatic filariasis: *Brugia pahangi* (BPAG_0000454501), and *W. bancrofti* (WBA_0000973501). The *Brugia timori* draft genome encodes a partial sequence with perfect homology to the last 72 amino acids of Bm18019 (BTMF_scaffold0002080 6441 to 6656). No homologous genes were identified in other organisms and the possibility for a unique role in the LF niche remains to be explored.

One limitation of this study was that the comparison was carried out with *B. malayi* instead of *L. loa* due to limited access to *L. loa* worm products. A lack of laboratory models and molecular reagents is a common challenge in filariasis research. The parasites cannot be cultured *ex vivo* and the only human filarial parasites whose lifecycle can be reproduced in the laboratory using rodent hosts are *B. malayi* and only recently *L. loa* (10). However, *B. malayi* is an appropriate model for understanding the AD12 glycoproteins in *L. loa*. The two organisms have a similar AD12 antigen profile by western blot and there was a high level of overlap between the AD12 glycoproteins in cross-reactive loiasis serum antigens and BmSOM. A second limitation is that the immunoprecipitation wash conditions prior to proteomics differed between BmSOM and BmES. The BmSOM was washed identical to the LoaCFAs (9), and it is possible that the BmSOM proteomics may have more false positives due to less stringent wash conditions compared to BmES, however, false positives were identified by the isotype control immunoprecipitation which used the same wash conditions.

Given the ubiquitous nature of AD12e in multiple parasite species, and the fact that a number of filarial worms including *L. loa, B. malayi* and *Mansonella* species have blood stages, it is remarkable that the FTS is functionally specific for Bancroftian filariasis. This is explained in part due to the unique stability of the Wb-CFA protein compared to AD12-glycoproteins from *Brugia* and *Loa* (7). We speculate that the rare occurrence of FTS-positive antigenemia in loiasis is a result of stochastic worm damage or death as opposed to regulated secretion. The banding pattern of *L. loa* antigens resembles the serum profile in Bancroftian filariasis after microfilaricidal treatment further arguing that worm death is responsible in both cases (25).

In summary, our data highlight the unique nature of Wb-CFA, compared to AD12e glycoproteins of other nematodes. The stability and persistence of Wb-CFA in serum has enabled its use as a functionally specific marker of Bancroftian filariasis and in turn led to the development of RDTs that expedited elimination of LF from many endemic areas. Ongoing efforts to clearly identify Wb-CFA, the AD12e structure, and to elucidate differences between Wb-CFA and cross-reactive loiasis antigens should facilitate the development of improved diagnostic tests for filarial infections.

## Supplementary Material

Table 1 **Supplementary Table 1 |** Complete list of AD12 epitope-containing proteins identified from *B. malayi*.

Table 3 **Supplementary Table 3 |** Raw lectin array data.

Data Sheet 1 **Supplementary Figure 1 |** *C. elegans* expresses AD12 reactive glycoproteins.
**Supplementary Figure 2 |** Bm18019 is a putative mucin.
**Supplementary Figure 3 |** PNGase F control reaction.

Table 2 **Supplementary Table 2 |** GO term analysis.

## Figures and Tables

**TABLE 1 |:** Top proteins found in immunoaffinity purified *B. malayi* antigen preparations.

No. Unique Peptides		Sequence Details		Sequence Analysis*			homolog found in Loa CFAs**
BmSOM	ES	Description	ID	MW (kDa)	O-glycosite	N-glycosite	
**BmSOM and ES**							
67	22	Hypothetical C-type lectin	Bm5701	74	115	2	-
51	2	Hypothetical immunoglobulin I-set domain containing protein	Bm10822b	712	147	4	Y
41	22	Molecular chaperone HtpG	Bm13653	83	5	3	Y
40	17	Galectin (lec-2)	Bm4277	32	0	0	Y
34	23	Heat shock 70kDa protein 1/8	Bm3611	70	6	3	Y
30	13	Glyceraldehyde 3-phosphate dehydrogenase	Bm5699a	36	1	0	Y
29	2	Alpha-1,4 glucan phosphorylase	Bm5931a	98	2	1	-
26	14	Hypothetical protein	Bm8327	39	112	1	-
26	7	Phosphoglycerate kinase	Bm13839	45	0	1	-
23	18	L-lactate dehydrogenase	Bm3339	38	3	0	Y
**BmSOM only**							
113	-	Aminopeptidase	Bm5654a	126	11	10	Y
47	-	Oxidoreductase	Bm2014	138	12	0	Y
37	-	Hypothetical PAN domain containing protein	Bm5611	210	109	5	Y
34	-	Filarial antigen Av33	Bm3738	26	13	0	Y
33	-	Hypothetical protein	Bm14333	69	47	2	-
33	-	Hypothetical protein	Bm14109	361	7	4	-
21	-	Hypothetical protein	Bm8924	55	19	2	-
17	-	Tubulin beta	Bm4733	50	0	1	Y
15	-	Hypothetical lipase family protein	Bm4899	59	86	1	-
**ES only**							
-	33	Myotactin form B	Bm4801a	536	125	3	Y
-	27	Triosephosphate isomerase (TIM)	Bm13880	27	0	0	-
-	13	Fructose-bisphosphate aldolase, class I	Bm5580	40	4	0	-
-	13	2,3-bisphosphoglycerate-independent phosphoglycerate mutase	Bm13317	57	2	0	-
-	10	14-3-3 protein beta/theta/zeta	Bm4259b	29	4	2	-
-	8	Hypothetical protein	Bm18019	53	106	0	-
-	8	Moesin/ezrin/radixin homolog 1	Bm1890	67	17	0	-
-	8	Heat shock protein 110kDa	Bm13663	92	11	2	Y
-	7	Larval allergen	Bm97	30	11	3	-
-	6	Hypothetical protein	Bm4209b	59	0	0	-
-	6	Oxidoreductase	Bm9132	44	0	1	-

* Predicted number of glycosylation sites according to NetOGlyc 4.0 and NetNGlyc 1.0.

** Hertz et al. (9).

**TABLE 2 |:** In-gel proteomics of AD12 glycoproteins from BmES.

Protein ID	No. Unique peptides	Description	Spectra		Predicted	Detected in other IPs		
			Band 1	Band 2	MW (kDa)	ES	BmSOM	LoaCFAs*
Bm11186	9	Myosin heavy chain-3	9	0	227	-	-	-
Bm8494	8	Heparan sulfate proteoglycan 2	10	0	375	-	-	-
Bm11627	7	Lin-5 interacting protein/Rootletin	7	0	255	-	-	-
Bm3307	6	Myosin heavy chain-4	6	0	225	-	Y	Y
Bm18019	3	Hypothetical protein	0	6	53	Y	-	-
Bm3162	3	Paramyosin	0	3	101	-	Y	Y
Bm5931a	2	Alpha-1,4 glucan phosphorylase	0	3	98	Y	-	-

* Hertz et al. (9).

## Data Availability

The original contributions presented in the study are included in the article/Supplementary Material. The mass spectrometry proteomics data have been deposited to the ProteomeXchange Consortium via the PRIDE partner repository with the dataset identifier PXD027003. Further inquiries can be directed to the corresponding author.
