# Supplementary material for: Brugia malayi Glycoproteins Detected by the Filariasis Test Strip Antibody AD12.1
Source: Front Trop Dis. Author manuscript; Available in PMC 2024 Jul 3. (PMC11220778; doi:10.3389/fitd.2021.729294)
Supplement: Data Sheet 1 — Supplementary Figure 1 | C. elegans expresses AD12 reactive glycoproteins. Supplementary Figure 2 | Bm18019 is a putative mucin. Supplementary Figure 3 | PNGase F control reaction. [file NIHMS2003410-supplement-Data_Sheet_1.pdf]

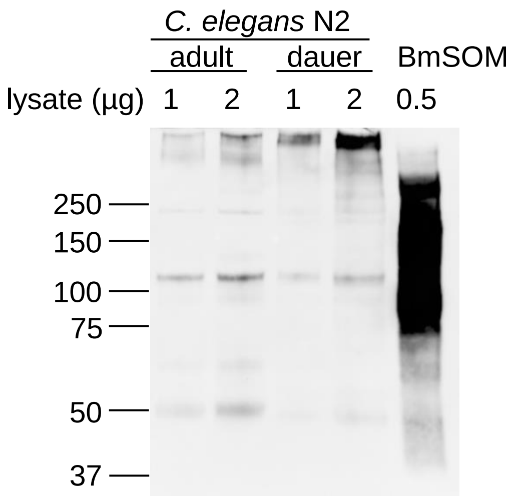

Figure S1: AD12.1 reactive proteins evident in the free-living nematode, *C. elegans.* Whole worm lysates from N2 strain adult or dauer stages were resolved by SDS-PAGE and immunoblotted with AD12.1. Adult female soluble antigens from *B. malayi* (BmSOM) served as a positive control.

A

```
MSQDSTSSTYRRPIFSGQVAMAYAVRTLLSWHTLTMSLKCLFAIFLASNAYPTLQKDGTA
NYNVKTESELHPIPLPVFPETSSLPEPETLTHMKVPDDSERYPSFPPLFPETSSPPEPET
STHMKVPDDSERYPSFPPLFPETSSPPEPETSTHMKVPDDSERYPIFPPLFPETSSPPEP
ETSTHMKVPDDSERYPSFPPLFPETSSPPEPETSTHMKVPDDSERYPSFPPLFPETSSPP
EPETSTHMKVPDDSERYPIFPPLFPETSSPPEPETSTHMKAPDDSERYPIFPPLFPETSS
PPEPETSTHMKAPDDSERYPIFPPLFPETSSSPEPETSSQLKLPSDSERHHIPSLIFPET
SSSPEPETSSQMKLPSDSERHHIPSLIFPETSSSPEPETSSQMKLPSDSERHHIPSLIFP
ETSSSPGLEKSTHMVIPSDFSTNYDIRTESEWNSYAKQFSVLPYFLNHPLLRR
```

B

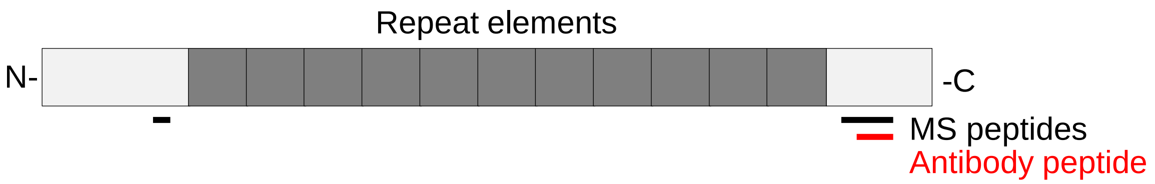

C

78	FPETSSLPEPETLTHMKVPDDSERYPSFPPL	108
109	FPETSSPPEPETSTHMKVPDDSERYPSFPPL	140
141	FPETSSPPEPETSTHMKVPDDSERYPIFPPL	171
172	FPETSSPPEPETSTHMKVPDDSERYPSFPPL	203
204	FPETSSPPEPETSTHMKVPDDSERYPSFPPL	235
236	FPETSSPPEPETSTHMKVPDDSERYPIFPPL	267
268	FPETSSPPEPETSTHMKAPDDSERYPIFPPL	299
300	FPETSSPPEPETSTHMKAPDDSERYPIFPPL	331
332	FPETSSSPEPETSSQLKLPSDSERHHIPSLI	363
364	FPETSSSPEPETSSQMKLPSDSERHHIPSLI	395
396	FPETSSSPEPETSSQMKLPSDSERHHIPSLI	427
428	FPETSSSP	435

Figure S2: Bm18019 is a putative mucin. (A) Predicted amino acid sequence of Bm18019 with identifying mass spec peptides indicated. **(B)** Scaled schematic of the Bm18019 protein. The majority of the protein is comprised of 11 repeat elements depicted as gray boxes. The location of mass spec peptides and the peptide used for antibody production is denoted by black and red bars, respectively. **(C)** Alignment of Bm18019 repeat elements with serine and threonine residues highlighted in yellow and cyan, respectively.

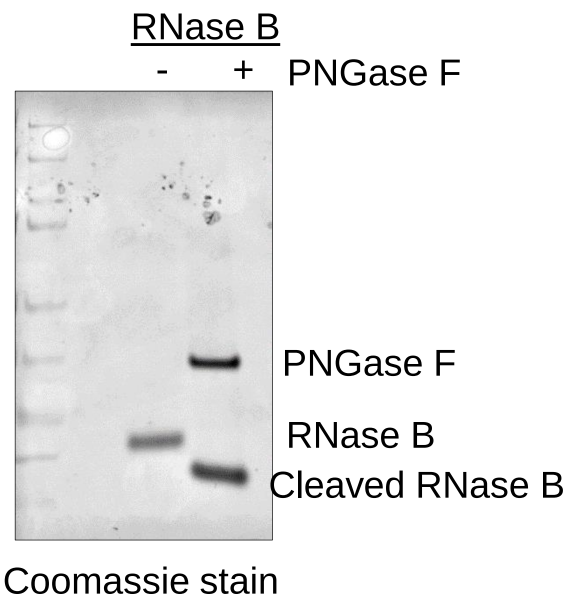

Figure S3. PNGase F positive control reaction. One microgram of RNase B (New England Biolabs) was treated with PNGase F for one hour at 37°C as per the manufacturer's protocol. The cleavage products were resolved by SDS-PAGE and stained with InstantBlue.
